# Particularities of urological complications in Crohn's disease: Report of two cases

**DOI:** 10.1016/j.amsu.2022.103903

**Published:** 2022-06-06

**Authors:** Taoufik Elabbassi, amine Bachar, Aziz Moufakkir, yassine El Berni, karim Yaqine, Mohamed Rachid Lefriyekh

**Affiliations:** a Visceral Surgery Department, University Hospital Center Ibn Rochd, Casablanca, Morocco; b Faculty of Medicine and Pharmacy, Hassan II University, Casablanca, Rue des Hôpitaux, Casablanca, Morocco

**Keywords:** Crohn's disease, Urological complication, Fistula, Surgery

## Abstract

Urological complications of Crohn's disease are rare, often asymptomatic and present a diagnostic problem. These complications are dominated by fistulas. The renal prognosis may be involved. We report an observation of two clinical cases with urological complications of Crohn's disease.

**Case 1:**

Patient aged 26 years, followed for Crohn's disease. He presented with right iliac fossa pain related to a collection responsible for right uretero-hydronephrosis. Renal scintigraphy objectified that it was a dumb right kidney.

**Case 2:**

Patient was 37 years old, with no history; he consulted for pollakiuria and pneumaturia. Surgical exploration showed the presence of a vesico-colic fistula. Histological examination of the fistula path was related to Crohn's disease.

**Conclusion:**

Urological complications of Crohn's disease are rare but can become serious, their diagnosis is difficult, sometimes these complications can be inaugural.

## Highlights

• Urological complications of Crohn's disease are rare.
• Present a diagnostic problem.
• Complications are dominated by fistulas.
• The renal prognosis may be involved.

## 1 Introduction

Crohn's disease is a chronic inflammatory bowel disease (IBD) that can affect the entire digestive tract from mouth to anus [1]. Some extra-digestive manifestations may accompany it, such as skin, ophthalmologic and joint manifestations [2]. Urological manifestations are less well described and often go unnoticed due to their asymptomatic nature in most cases [2]. We report on two clinical cases that illustrate the particularities of these urological complications**.** This work has been reported in line with the SCARE criteria [3]

## 2 Case 1

A 26 year old patient. Two years ago he underwent ileo-caecal resection with immediate restoration of continuity, following an occlusive syndrome, on an inflammatory stenosis of the last ileal loop. Histological examination of the specimen showed Crohn's disease in acute inflammatory flare. The patient was put on medical treatment with intestinal anti-inflammatory drugs. After six months of the operation, the patient presented with lumbar and right iliac fossa pain, the clinical examination was unremarkable. The abdominal CT scan showed a collection in the right iliac fossa measuring 70mm × 28mm, which included the right lumbar ureter and caused upstream pyelo-caliceal dilatation with thickening of the adjacent bowel.

**Fig. 1:**
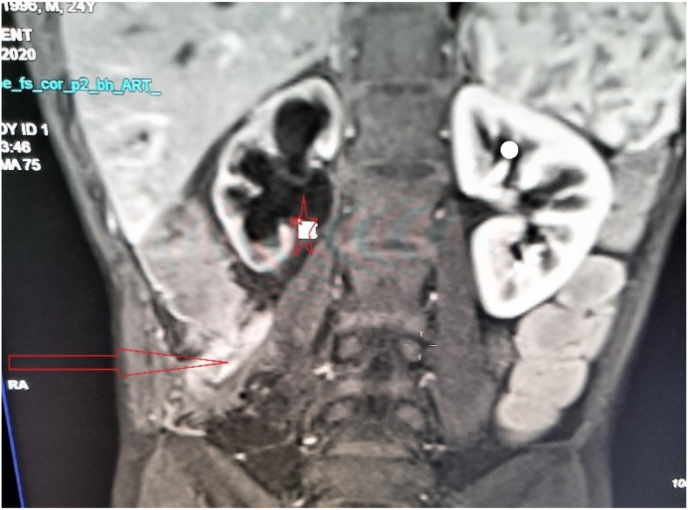
ENTERO-MRI image showing a retroperitoneal collection in the right flank (arrow) with right ureterohydronephrosis (star).

**Fig. 2:**
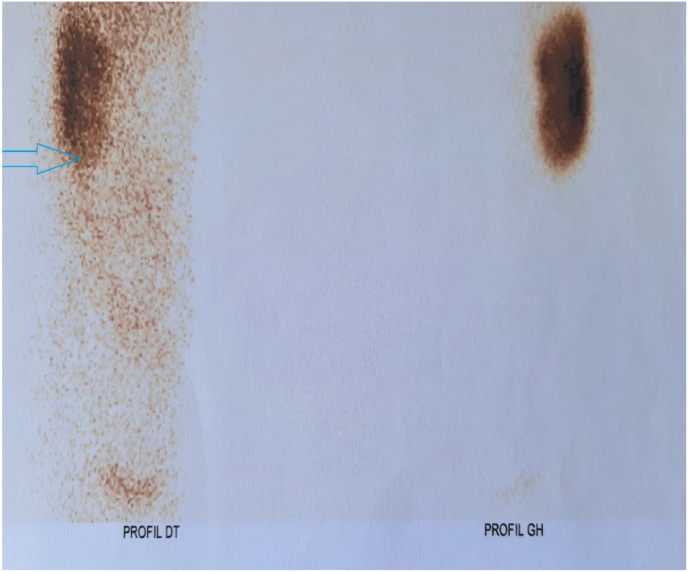
DMSA static renal scan showing collapsed right renal function (arrow).

The MRI scan showed a retroperitoneal collection in the right flank measuring 49 × 19 mm and extending over 57 mm, communicating with the colon, suggesting a blind fistula complicated by right ureterohydronephrosis (Fig. 1). Static renal scintigraphy with DMSA showed a collapsed right renal function estimated at less than 5% (Fig. 2).

**Fig. 3:**
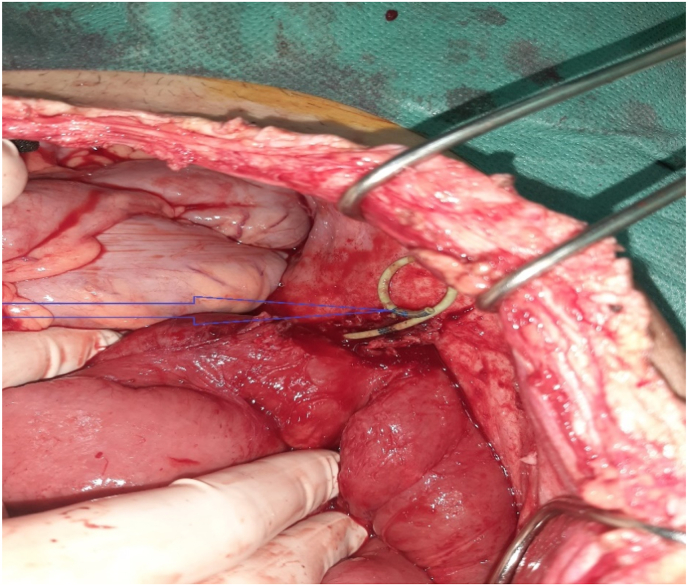
Image showing the double J probe through the ureteral fistula (arrow).

Creatinine was normal at 10.8 mg/L, white blood cells were elevated at 16170 elements/mmᶾ and CRP at 320 mg/L. The patient had a double J tube mount with failed radiological drainage. The patient was scheduled for surgical evacuation of the collection with a right nephrectomy. Surgical exploration showed the presence of a purulent retroperitoneal collection related to a ureteral fistula and the old thickened and stenotic terminal ileo-colic anastomosis. The double J catheter was located intraperitoneally (Fig. 3). The patient underwent ileocolic resection with removal of the old anastomosis, restoration of digestive continuity and right nephrectomy with closure of the distal ureter. The postoperative course was simple.

## 3 Case 2

**Fig. 4:**
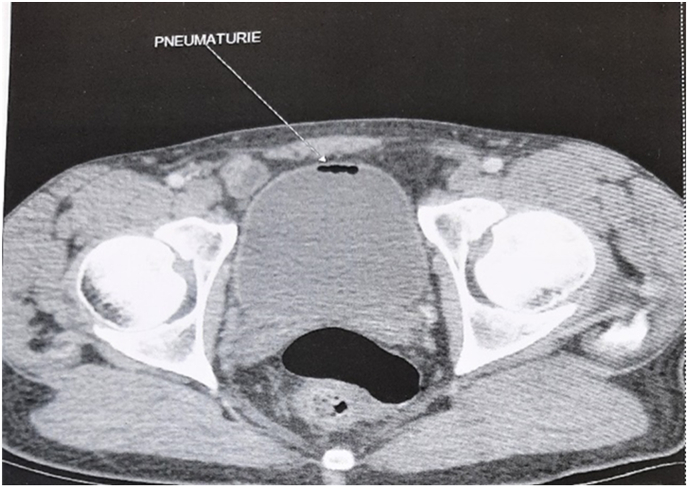
Abdominal CT scan showing bladder pneumaturia.

A 38 year old patient with no previous history. He consulted for pollakiuria complicated by pneumaturia evolving for fifteen days with alternating episodes of diarrhea and constipation. Cytobacteriological examination of the urine showed a multi-sensitive Escherichia Coli urinary infection. The patient was put on antibiotic therapy and underwent cystoscopy, which came back without any particularity. Abdominal and pelvic CT scan with opacification showed bladder pneumaturia without visualization of a urodigestive fistula (Fig. 4). Colonoscopy showed a diverticular sigmoid. Biopsies were inflammatory without specificity. Surgical exploration revealed a fistula between the posterior bladder wall and the sigmoid loop. He underwent disconnection of the fistula, biopsy of the edges of the fistula orifices at the colonic and bladder level, suture of the bladder wall and lateral sigmoid colostomy. Histological examination showed Crohn's type inflammatory disease. The postoperative course was simple. A restoration of colonic continuity was performed outside of the inflammatory flare-ups.

## 4 Discussion

Crohn's disease is a frequent cause of urodigestive fistula (5–10%), which may be inaugural [4]. Ileo-vesical fistula is the most common (64%), colo-vesical fistula (21%), recto-vesical fistula (8%) and ureterodigestive fistula (8%) are exceptional [5,6]. The anatomical arrangement of the bladder as an intraperitoneal dome explains the high rate of fistula in the bladder. The ureter is retroperitoneal, protected by the posterior parietal peritoneum. Our patient was initially operated on for an ileo-caecal resection requiring an opening of the posterior peritoneum.

The main symptoms of a vesico-digestive fistula are dysuria (68%), pneumaturia (64%), urinary tract infections (32%) and faecaluria (28%) [7]. The most useful tests for diagnosis are cystoscopy (74%) and CT scan (52%). Visualization of the fistula on cystography is not always easy to obtain [7]. In our patient the fistula was visualised by colonoscopy. Surgical disconnection was an option to medical treatment as our patient had no histological evidence. Otherwise, treatment with anti-TNF-alpha may allow fistula closure by treating the underlying inflammation [8].

Intra-abdominal or pelvic abscesses occur in 10–30% of patients followed for Crohn's disease. An abscess is suspected in any febrile abdominal pain, or pain associated with an intense biological inflammatory syndrome [9]. Our patient presented with lumbar abdominal pain and pain in the right iliac fossa. CT scan is the gold standard [9]. Treatment is drainage, preferably radiological [9]. The particularity of our patient was the impact on the upper limb by compression and fistulisation of the right ureter, resulting in a non-functional right kidney requiring its removal.

## 5 Conclusion

Urodigestive fistulas remain a rare complication compared to other extra-digestive manifestations. The bladder is the organ most affected. Ureteral involvement is exceptional. The treatment of choice remains surgical.

## Ethical approval

I declare on my honor that the ethical approval has been exempted by my establishment.

## Sources of funding

None.

## Author contribution

Moufakkir Aziz: Corresponding author writing the paper and operating surgeon, Bachar Amine: writing the paper and operating surgeon, El Abbassi Taoufik: writing the paper and operating surgeon, El berni Yassine: writing the paper, Yaqine karim: study concept, Mohamed Rachid Lefriyekh: correction of the paper.

## Declaration of competing interest

The authors declare having no conflicts of interest for this article.

## Trial registration number

None.

## Guarantor

Moufakkir aziz.

## Consent

Written informed consent for publication of their clinical details and/or clinical images was obtained from the patient. A copy of the written consent is available for review by the Editor-in-Chief of this journal on request.

## Provenance and peer review

Not commissioned, externally peer-reviewed.
